# Transperineal Prostate Biopsy Targeted by Magnetic Resonance Imaging Cognitive Fusion

**DOI:** 10.3390/diagnostics13081373

**Published:** 2023-04-08

**Authors:** Petru Octavian Drăgoescu, Andrei Ioan Drocaș, Alice Nicoleta Drăgoescu, Vlad Pădureanu, Andrei Pănuș, Andreea Doriana Stănculescu, Mihai Alexandru Radu, Lucian Mihai Florescu, Ioana Andreea Gheonea, Cecil Mirea, George Mitroi

**Affiliations:** 1 Department of Urology, University of Medicine and Pharmacy of Craiova, 200349 Craiova, Romania; 2 Department of Anesthesiology and Intensive Care, University of Medicine and Pharmacy of Craiova, 200349 Craiova, Romania; 3 Department of Internal Medicine, University of Medicine and Pharmacy of Craiova, 200349 Craiova, Romania; 4 Department of Urology, Emergency Clinical County Hospital of Craiova, 200642 Craiova, Romania; 5 Department of Radiology and Medical Imaging, University of Medicine and Pharmacy of Craiova, 200349 Craiova, Romania; 6 Department of Surgery, University of Medicine and Pharmacy of Craiova, 200349 Craiova, Romania

**Keywords:** prostate cancer, cognitive fusion biopsy, systematic biopsy, magnetic resonance imaging

## Abstract

Prostate cancer is among the most frequently diagnosed cancers and a leading cause of cancer-related death in men. Currently, the most reliable and widely used imaging test for prostate cancer diagnosis is multiparametric pelvic magnetic resonance imaging (mpMRI). Modern biopsy techniques are based on the computerised merging of ultrasound and MRI images to provide better vision during the biopsy procedure (Fusion Biopsy). However, the method is expensive due to high equipment cost. Cognitive fusion of ultrasound and MRI images has recently emerged as a cheaper and easier alternative to computerised fusion. The aim of this prospective study is to perform an in-patient comparison of the systematic prostate biopsy procedure (SB) vs. cognitive fusion (CF) guided prostate biopsy method in terms of safety, ease of use, cancer detection rate and clinically significant cancer detection. We enrolled 103 patients with suspected prostate cancer that were biopsy naive, with PSA > 4 ng/dL and PIRADS score of 3, 4 or 5. All patients received a transperineal standard 12–18 cores systematic biopsy (SB) and a four-cores targeted cognitive fusion (CF) biopsy. Following the prostate biopsy, 68% of the patients were diagnosed with prostate cancer (70/103 patients). SB diagnosis rate was 62% while CF biopsy was slightly better with a 66% rate. There was a significant 20% increase in clinically significant prostate cancer detection rate for the CF compared to SB (*p* < 0.05) and a significant prostate cancer risk upgrade from the low to the intermediate risk category (13%, *p* = 0.041). Transperineal cognitive fusion targeted prostate biopsy is a straightforward biopsy method that is easy to perform and is a safe alternative to standard systematic biopsy with improved significant cancer detection accuracy. A combined targeted and systematic approach should be used for the best diagnostic results.

## 1. Introduction

Prostate cancer is among the most frequently diagnosed cancers and a leading cause of cancer-related death in men [1]. Clinical suspicion is based on abnormal digital rectal examination (DRE) and/or elevated serum levels of PSA (Prostatic Specific Antigen). DRE alone detects prostate cancer in 18% of patients regardless of PSA levels [2]. PSA is an organ-specific marker, but not cancer-specific. High levels of PSA indicate a greater likelihood of prostate cancer but may also be due to prostatitis or benign prostatic hyperplasia (BPH). PSA is considered a better predictor of cancer than DRE or transrectal ultrasound (TRUS) [3].

Ultrasound-guided transrectal systematic prostate biopsy has been the standard diagnostic technique for prostate cancer for quite a long time, but it is still far from being perfect due to a significant complication rate [4] as well as low accuracy and significant under-diagnosis rates. Transperineal prostate biopsy has a lower risk of complications while having similar accuracy with the transrectal biopsy in detecting clinically significant prostate cancer [5,6].

Currently, the most reliable and widely used imaging test for prostate cancer diagnosis is the multiparametric pelvic magnetic resonance imaging (mpMRI) and the Prostate Imaging-Reporting and Data System (PIRADS) Score v2.0 [7,8].

Modern prostate biopsy techniques are based on the computerised 3D merging of the ultrasound and MRI images to provide better vision during the procedure (Fusion Biopsy) [9,10]. However, the method is expensive due to high equipment costs. Cognitive fusion of ultrasound and MRI images has recently emerged as a cheaper and easier alternative to computerised fusion biopsy [11].

Prostate cancer grade reporting has been performed using the Gleason score system since 1977 [12]. It has been widely used and constantly updated until 2005 when the International Society of Urological Pathology (ISUP) convened a consensus conference where they made significant changes to Gleason score reporting [13]. The current ISUP score was first introduced in 2014 as a new and improved grade grouping system [14] where all cases with Gleason scores ≤ six are classified as grade one or non-clinically significant that can be followed by active surveillance only. The term ‘clinically significant’ prostate cancer is currently used to differentiate cancers that may lead to morbidity or death and therefore require active treatment from those that do not [15].

The aim of this study is to compare the systematic prostate biopsy (SB) approach with the cognitive fusion (CF) guided prostate biopsy method in terms of safety, ease of use, overall cancer detection rate as well as clinically significant cancer detection (ISUP ≥ two or Gleason score ≥ seven).

## 2. Materials and Methods

The study was conducted as a prospective in-patient comparison study, with patients acting as their own control. Between June and November 2022, a total of 138 patients with suspected prostate cancer (abnormal DRE and/or elevated PSA) that underwent prostate mpMRI were screened for the study. Patients with no or incomplete mpMRI reports, those with PIRADS scores 1 or 2 and subjects not willing to participate were excluded. Two patients that were initially been included in the study were eventually dropped due to pathology finding non-adenocarcinoma tumours after biopsy. We finally enrolled 103 patients with suspected prostate cancer that were biopsy naive and had abnormal DRE and/or PSA > 4 ng/dL as well as a prostate mpMRI report with PIRADS v2.0 score of 3, 4 or 5.

Age, body weight, lifestyle, medical history as well as IPSS were documented. Clinical exams including DRE, safety routine blood tests, abdominal ultrasound and TRUS with prostate volume calculation were performed for all subjects. PSA value, PSA density and PIRADS score were also recorded. All patients received a transperineal standard 12–18 cores systematic biopsy (SB) followed by a 4-cores targeted transperineal cognitive fusion (CF) biopsy performed by a different operator after analysing the mpMRI images and locating the tumour together with the radiology specialist using the PIRADS v2 prostate sector map [8]. All procedures were performed under antibiotic prophylaxis with quinolones using local, loco-regional anaesthesia or light sedation depending on patient and/or physician preference.

For the transperineal ultrasound-guided biopsy, we used a Toshiba/Canon Aplio 500 ultrasound system and the high-performance ultrawideband endorectal biplane transducer PVL-715RST with enhanced Precision+ technology and 170° (convex) and 56 mm (linear) wide field of view. High-definition images obtained with this transducer provided easy identification of the prostate zones on the prostate sector map as well as improved operator orientation during the cognitive part of the biopsy procedure. Disposable Bard MaxCore 18G biopsy guns and transducer-mounted adjustable needle bracket were used for transperineal prostate biopsy.

The number of biopsy cores, prostate cancer type, Gleason score, ISUP score, laterality, TNM and the number of positive biopsies were recorded for all patients as well as procedure side effects or complications. Biopsy was considered positive if prostate carcinoma with a Gleason score ≥ 6 was diagnosed. Clinically significant cancer was considered for patients with a Gleason score ≥ 7 or ISUP score ≥ 2 as per current guidelines. Prostate cancer risk groups were classified following the D’Amico criteria [16]:

- Low risk: PSA ≤ 10 ng/mL and Gleason score 6 or ISUP 1 and T ≤ 2a;
- intermediate risk: PSA 10–20 ng/mL or Gleason score 7 or ISUP score 2–3 or T2b;
- high risk: PSA ≥ 20 ng/mL or Gleason score ≥ 8 or ISUP 4–5 or T ≥ 2c.

Results were reported and analysed by study team members using MSExcel and SPSS version 20 software. Data normality was assessed by the Kolmogorov-Smirnov test. For normally distributed data comparison we used the Student *t*-test while the Mann–Whitney U-test was used as a nonparametric test. A chi-square test (χ2) was performed to test whether there is a connection between the use of cognitive fusion biopsy and improved overall prostate cancer diagnosis as well as better detection of clinically significant cancer.

## 3. Results

Patient ages ranged from 51 to 79 years with an average of 66.7 ± 6.5 years, while average BMI was 28.6 ± 3.8. Current cigarette smokers were 12 patients (11.6%) while 46.6% had a chronic alcohol consumption habit. Medical history revealed hypertension, obesity and type II diabetes mellitus as the most frequent associated conditions (46.6%, 34.9% and 25.2%, respectively). No significant routine blood test abnormalities were identified.

Lower urinary tract symptoms (LUTS) were present in most patients (95.1%) and IPSS ranged from 2 to 23, with an average of 14.1 ± 5.3. DRE was normal (T1) in 71 patients (68.9%), while 32 patients had abnormal DRE classified as T2 (31.1%). The right prostate lobe was abnormal in 14 patients (43.7%) and the left lobe in 16 patients (50%), while two patients had both lobes involved (6.25%). Prostate volume measured by transrectal ultrasound (TRUS) ranged from 18 to 69 cc with an average of 44.7 ± 11.3 cc. PSA values for study patients ranged from 4.2 to 27 ng/mL with an average value of 11.4 ± 4.5 ng/mL, and PSA density was 0.24 ± 0.6. Multiparametric MRI revealed PIRADS 3 score lesions for 27 patients (26.2%), PIRADS 4 score in 39 (37.8%) and PIRADS 5 score for 37 patients (36.0%). No significant correlations were identified between the PIRADS scores and DRE, PSA or prostate volume.

**Figure 1:**
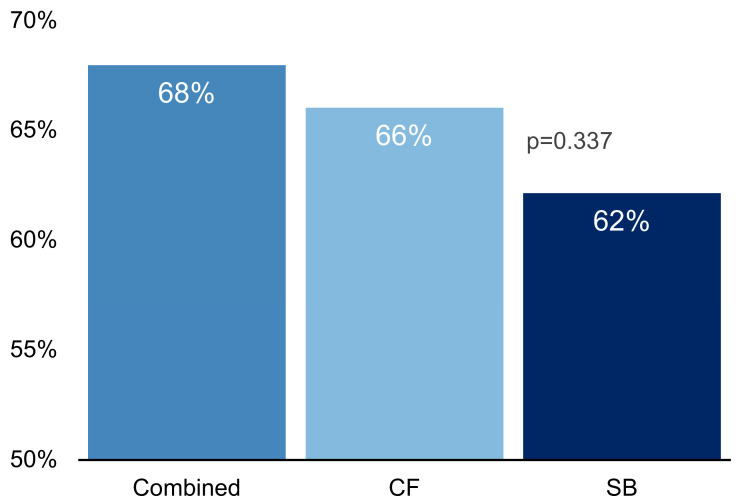
Diagnostic rate of transperineal ultrasound guided prostate biopsy types reveals that the combined method has the highest overall prostate cancer diagnostic rate, followed by CF and SB. No statistically significant difference was found between CF and SB (*p* = 0.337, chi-square test). CF = cognitive fusion biopsy, SB = systematic biopsy.

Following the prostate biopsy, 68% of the subjects were diagnosed with Gleason ≥ 6 prostate cancer (70/103 patients). Systematic prostate biopsy (SB) identified prostate cancer in 64 patients (62.1%) while with the cognitive fusion (CF) guided prostate biopsy was better with a diagnosis rate of 66% (68/103 patients), but not statistically significant (*p* = 0.337). There were two patients identified by SB alone and six by CF alone, while the remaining 62 patients (60%) were diagnosed with prostate cancer by both biopsy methods (Figure 1). We, therefore, note that although CF seems better than SB, the highest overall prostate cancer diagnostic rate was achieved for combined cognitive fusion and systematic biopsy techniques with no statistically significant diagnosis advantage identified for the CF technique.

Most of the patients with prostate cancer had localised T1c disease (38 subjects, 54%) while the rest had palpable T2a, T2b or T2c cancer (32 subjects, 46%), with no significant difference between the two groups (*p* = 0.558).

**Figure 2:**
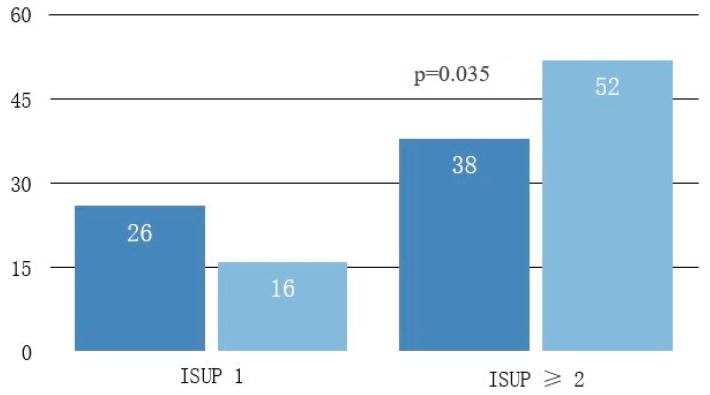
Pathology ISUP score significant increase by using CF vs. SB led to the diagnosis of more clinically significant cancer and upgraded patient prognostic group. CF = cognitive fusion biopsy, SB = systematic biopsy.

Among the 70 patients diagnosed with prostate cancer in our study, there were 16 patients with ISUP 1/Gleason 6 score (22.9%) and 54 patients (77.1%) with clinically significant cancer (Gleason score ≥ 7 or ISUP score ≥ 2). SB alone identified clinically significant ISUP ≥ 2 cancer in 38 patients (59.3%) while CF diagnosed 14 additional patients (52 patients, 76.4%, *p* = 0.035, chi-square test), which represent 20% from the total of 70 patients diagnosed with prostate cancer (Figure 2).

Similarly, for the ISUP 1 group, CF identified 10 patients less than SB alone. The four-subject difference between the two is due to CF diagnosing four additional ISUP ≥ 2 patients. Regarding the other ISUP score groups, we noted an upgrade by CF for all ISUP categories: five patients with ISUP 2, one with ISUP 3, two with ISUP 4 and six with ISUP 5, but none were statistically significant (Table 1).

We, therefore, noted that the prostate cancer risk was upgraded by cognitive fusion biopsy for 14 (20%) of the 70 subjects in our study. This is equivalent to every one in five patients potentially missing adequate prostate cancer treatment if SB only was performed.

Regarding D’Amico prostate cancer risk groups, we identified 11 patients in the low-risk group (15.7%), 43 in the intermediate group (61.4%) and 16 in the high-risk group (22.9%). Cognitive fusion biopsy provided a significant risk upgrade with nine patients being moved from the low to the intermediate risk category (11 vs. 20 subjects, 13%, *p* = 0.041) and four other patients being diagnosed with high instead of intermediate risk cancer (12 vs. 16, 6%) (Table 2).

**Figure 3:**
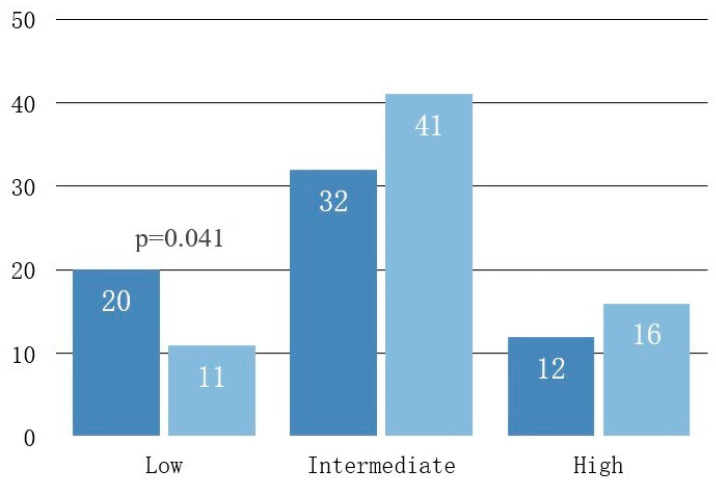
Risk group change by CF vs. SB led to the diagnosis of more aggressive cancer. CF = cognitive fusion biopsy, SB = systematic biopsy.

Biopsy procedure adverse events were rare (8.7%) and mild: self-limiting mild haematuria (five patients, 4.8%), prostatitis (three patients, 2.9%), urinary retention (two patients, 1.9%) and perineal hematoma (one patient, 1%). No additional complications were related to CF biopsy use (Figure 3).

## 4. Discussion

While the software-guided fusion prostate biopsy technique relies on the computerised merging of the MRI and ultrasound images, the cognitive fusion method is based on the ability of the trained human brain to accurately locate an item based on an accurate description, or after visualising a detailed map. The term cognitive is defined as connected with thinking or conscious mental processes. Cognitive procedures have actually been performed in medicine ever since the introduction of medical imaging during the early 20th century. The cognitive analysis of medical images (X-ray, CT, ultrasound, MRI, etc.) helped clinicians to better and more accurately identify and treat whatever pathology with which they were dealing.

The cognitive fusion prostatic biopsy procedure, therefore, involves a mental overlapping of the static mpMRI images with the live ultrasound image used during the prostate biopsy. It requires that the operator masters prostate anatomy as well as both ultrasound and MRI imaging analysis and, if possible, works together with a specialised radiologist to obtain the best location of the suspected tumour. While arguably more challenging for the biopsy operator than the computerised fusion procedure, cognitive fusion has the obvious advantage of a significantly lower price.

The actual cognitive fusion procedure details were described by Verma et al. in 2017 along with other types of prostate biopsy methods. They state that procedure accuracy depends on the skill of the operator in translating the targets identified at MRI to the anatomy recognised at transrectal ultrasound as the imaging planes of MRI sections differ from transrectal ultrasound [17]. We addressed these issues in our study by constantly working with the radiologist and using the biplane transperineal biopsy approach which provides ultrasound images that are similar to those obtained from MRI. The 4-core cognitive fusion biopsy was also chosen as it was shown to be optimal in recent studies [18]. For improved targeted biopsy accuracy we used the PIRADS v2 criteria and prostate sector map described by Weinreb et al. in 2015 that divides the prostate into 39 zones and is accompanied by an actual graphic map that was used as an additional tool during the fusion biopsy [19]. Since its introduction, it has gradually become the standard MRI prostate tumour reporting in our institution and currently includes the latest updates from 2019 [8]. We believe that the use of the transperineal approach guided by biplane transrectal ultrasound for prostate biopsy is the best approach for the cognitive fusion procedure as it produces image orientation that is very similar to that provided by the mpMRI analysis both in the transversal and sagittal plane. The transperineal approach also provides better access to the anterior part of the prostate, which is usually difficult to safely reach during a classic transrectal biopsy. We used the transversal plane for anteroposterior and lateral orientation and the sagittal plane for the cranio-caudal orientation (base/apex) and the biopsy procedure as the biopsy needle bracket is oriented in this plane.

Following mpMRI, most of the patients in our study group had PIRADS 4 and 5 score lesions (74%) while only 26% had PIRADS 3 lesions which is similar to other reports [20,21,22]. However, in contrast to other studies, we found no significant correlation between the PIRADS score and PSA values.

Overall prostate cancer diagnostic rate for transperineal prostate biopsy in our study was 68% when combining the results of both biopsy methods. This rate was better than any of the individual methods (62% SB and 66% CF). CF identified four more cancer patients than SB, but the difference was not statistically significant (*p* = 0.337). These results are consistent with the findings of other similar studies that found no difference in the overall cancer detection rate between the two methods [23,24]. However, our data showed an important 20% increase in clinically significant cancer detection (Gleason score ≥ 7 or ISUP score ≥ 2) by using cognitive fusion biopsy. These findings are in line with the reports of most recent studies. Ahmet et al. similarly showed in a larger study that included 740 patients that if TRUS-biopsies were directed by mpMRI findings, up to 18% more cases of clinically significant cancer might be detected compared with the standard TRUS-biopsy [25]. They concluded that mpMRI can reduce the over-diagnosis of clinically- nsignificant prostate cancer and improve the detection of clinically significant cancer. Likewise, another study from 2020 by Miah et al. found that non-targeted prostate biopsy cores had a low yield of clinically significant prostate cancer and a high yield of clinically insignificant cases while the image-fusion targeted-biopsy-approach had opposite results [26]. The 2018 UK-based PRECISION global trial by Kasivisvanathan et al. showed that detection of insignificant disease was 13% higher in the systematic biopsy comparator arm (22% vs. 9%) and that mp-MRI targeted prostate biopsy improved the significant cancer detection rate by 16% [27]. We obtained similar results in our study regarding prostate cancer risk groups. This was expected as the criteria for low-risk cancer and clinically insignificant cancer overlap significantly. There was a risk group upgrade from low to intermediate risk for 13% of our patients.

Another large study performed by Ahdoot et al. in 2020 included 2103 patients that underwent MRI-Targeted, systematic, and combined biopsy for prostate cancer. They found the best diagnosis rate for combined targeted and fusion biopsy (62.4%). MRI-Targeted biopsy had a lower cancer detection rate for grade group 1 cancers and higher for grade groups 3–5. They found a 10% diagnostic rate increase and 22% risk group upgrade for the combined biopsy [28]. However, they used computerised fusion which is superior to cognitive fusion, but more expensive.

Regarding the comparison with computerised fusion, several recent studies have shown the superiority of software fusion biopsy over cognitive fusion especially regarding clinically significant cancer [29,30], while other authors could not find any significant difference between the two fusion strategies, especially for larger mpMRI lesions [31,32]. Furthermore, Hamid et al. compared the two fusion strategies in the SmartTarget Biopsy Trial and found no difference between them while showing that they should be combined to obtain the best detection rate for clinically significant prostate cancer [33]. A comprehensive review by Sugano et al. evaluated the current literature regarding different modalities of mpMRI-targeted biopsy for the detection of prostate cancer. They concluded that cognitive fusion targeted biopsy (COG-TB) has comparable detection rates to software fusion but is operator-dependent and may have reduced accuracy for smaller lesions [34].

Summarising our results as well as all this evidence we may conclude that while cognitive fusion biopsy may be slightly better than systematic biopsy in detecting overall prostate cancer in larger patient groups, it is superior at identifying clinically significant cancer subjects so that it helps up to one in five patients to avoid missing adequate prostate cancer treatment. However, the cognitive fusion procedure still has highly variable results probably due to operator dependency, registration and reporting differences, as well as biopsy approach or equipment variability, so the combination with systematic biopsy should still be used for best diagnosis results. Computerised fusion adds precision especially in smaller lesions, so, depending on procedure costs, it should probably be used in these cases in addition to cognitive fusion.

The limitations of our study include the lack of comparison to other fusion biopsy types like computerised or in-bore biopsy. We performed the study analysing results from a limited patient sample and used in-patient comparison instead of randomised groups. It would therefore be safe to assume that a larger patient sample and the use of separate randomised patient groups would significantly improve the results. Regarding clinically significant criteria, all Gleason 6/ISUP 1 patients were considered clinically insignificant irrespective of the positive biopsy cores number and tumour percentage. We did not stratify patients based on MRI lesion diameter or number as there is no consensus on that. However, all prostate cancer patients were subsequently clinically assessed by their respective urologists and treatment options were presented according to current guidelines. Unfortunately, due to variable treatment options available for prostate cancer patients and a lack of complete definitive pathology data, we could not compare all our biopsy results to radical prostatectomy specimens. We intend to approach most of these technical issues in a future larger randomised study.

## 5. Conclusions

Transperineal cognitive fusion targeted prostate biopsy is a straightforward biopsy method that is easy to perform and is a safe alternative to standard systematic biopsy with improved significant cancer detection accuracy. A combined targeted and systematic approach should be used for the best diagnostic results.

## Figures and Tables

**Table 1:** Pathological ISUP and Gleason scores for patients diagnosed with prostate cancer in our study (number, percentage % of the specific group). CF = cognitive fusion biopsy, SB = systematic biopsy, Combined = combination between SB and CF methods. * chi-square test.

ISUP Score	Gleason Score	SB (*n* = 64)	CF (*n* = 68)	Combined (*n* = 70)	Change SB vs. CF	* *p* = SB vs. CF
1	3 + 3	26 (40.6%)	16 (29.4%)	16 (22.9%)	−10 (14.3%)	*-*
2	3 + 4	12 (18.8%)	17 (25%)	17 (24.3%)	5 (7.1%)	*p* = 0.386
3	4 + 3	9 (14.1%)	10 (14.7%)	11 (15.7%)	1 (1.4%)	*p* = 0.916
4	4 + 4, 3 + 5	11 (17.1%)	13 (19.1%)	14 (20.0%)	2 (2.8%)	*p* = 0.773
5	4 + 5, 5 + 4, 5 + 5	6 (9.4%)	12 (17.6%)	12 (17.1%)	6 (8.5%)	*p* = 0.166
≥2	≥7	38 (59.4%)	52 (76.4%)	54 (77.1%)	14 (20.0%)	*p* = 0.035

**Table 2:** Distribution of D’Amico risk groups for patients diagnosed with prostate cancer in our study (number, percentage % of the specific group). CF = cognitive fusion biopsy, SB = systematic biopsy, Combined = combination between SB and CF methods. * chi-square test.

D’Amico Risk Group	SB (*n* = 64)	CF (*n* = 68)	Combined (*n* = 70)	Change SB vs. CF	* *p* = SB vs. CF
Low	20 (31.2%)	11 (16.2%)	11 (15.4%)	−9 (12.9%)	*p* = 0.041
Intermediate	32 (50%)	41 (60.3%)	43 (61.4%)	9 (12.9%)	*p* = 0.234
High	12 (18.8%)	16 (23.5%)	16 (22.9%)	4 (5.7%)	*p* = 0.502

## Data Availability

Not applicable.
